# A Review on Canine and Feline Prostate Pathology

**DOI:** 10.3389/fvets.2022.881232

**Published:** 2022-05-26

**Authors:** Chiara Palmieri, Carlos Eduardo Fonseca-Alves, Renee Laufer-Amorim

**Affiliations:** ^1^School of Veterinary Science, The University of Queensland, Gatton, QLD, Australia; ^2^School of Veterinary Medicine and Animal Science, Sao-Paulo State University-UNESP, Botucatu, Brazil

**Keywords:** dog, cat, prostate, pathology, review

## Abstract

Prostatic diseases are very common in male dogs, accounting for 3–10% of cases submitted to the veterinary practitioners. Commonly reported canine prostatic disorders include prostatic hyperplasia, prostatitis, prostatic cysts and prostatic carcinoma. However, clinical signs may be non-specific, or many cases are asymptomatic, thus leading to a difficult estimation of the actual prevalence of clinical cases. On the other side, because of the rare occurrence of prostate disease in cats, very little is known about pathogenesis, diagnostic approaches and treatment. The goal of this review is to provide detailed clinical and pathological overview of the feline and canine prostatic pathology, including the most up-to-date classification systems and histological findings. Emphasis is places on gross, cytological and histological features that are critical to reach a definitive diagnosis for a proper treatment and prognosis.

## Introduction

Diseases of the prostate are common in older male dogs, while occasionally reported in cats. The main challenge from a clinical perspective is the overlap of clinical signs referring to dysfunctions of the urinary and/or intestinal tract that may be observed in the majority of the disease processes. Moreover, some cases may be asymptomatic and go unnoticed or different lesions can be present simultaneously. It has been estimated that 75.6% of dogs that die of disease unrelated to the prostate have however prostatic disorders at post-mortem examination (1).

This means that estimating the prevalence of these disorders is quite difficult. This is particularly obvious when examining prostatic lesions in cats, while in dogs it is well known that some disorders (e.g., prostatic hyperplasia) accounts for the majority of cases of prostatic diseases, followed by prostatitis, tumors and squamous metaplasia, although a combination of different disease processes is very common (e.g., prostatitis concurrent with prostatic hyperplasia).

This article provides a comprehensive overview of prostatic lesions in dogs and cats, covering common diseases affecting this accessory gland of the male reproductive tract, as well as updates and terminologies that have been proposed in the last decade. However, it should be taken into consideration that the information reported by the previous and current literature may not be a real representation of the prevalence of different prostatic disorders due to the paucity of available data, the different frequency of castration in the country of origin of the studies that may influence the incidence of hormone-induced prostatic atrophy and the perception of a higher incidence of prostatic tumors in castrated dogs and sensitivity of the diagnostic procedures of choice.

## Prostate Anatomy and Histology

The prostate is the only accessory sex gland of the canine male reproductive tract, while cats possess both prostate and bulbourethral gland. The canine prostate is an ovoid-shaped, bilobed organ that completely envelopes the proximal portion of the urethra close to the neck of the bladder (2). The feline prostate is more caudally placed than in dogs, located 2–3 cm from the urinary bladder, behind the cranial border of the pelvic symphysis under the ventral wall of the rectum (3). It covers the urethra only dorsally and laterally (4). The canine prostate is enveloped by a fibromuscular capsule that receives smooth muscle fibers from the wall of the urinary bladder. The gland has a dorso-medial sulcus and a median septum that divides the prostate into right and left lobe. Each lobe is further separated into lobules by capsular trabeculae. The vas deferens enters the cranio-dorsal surface of each prostate lobe, ending up in the urethra by the colliculus seminalis (2).

**Figure 1:**
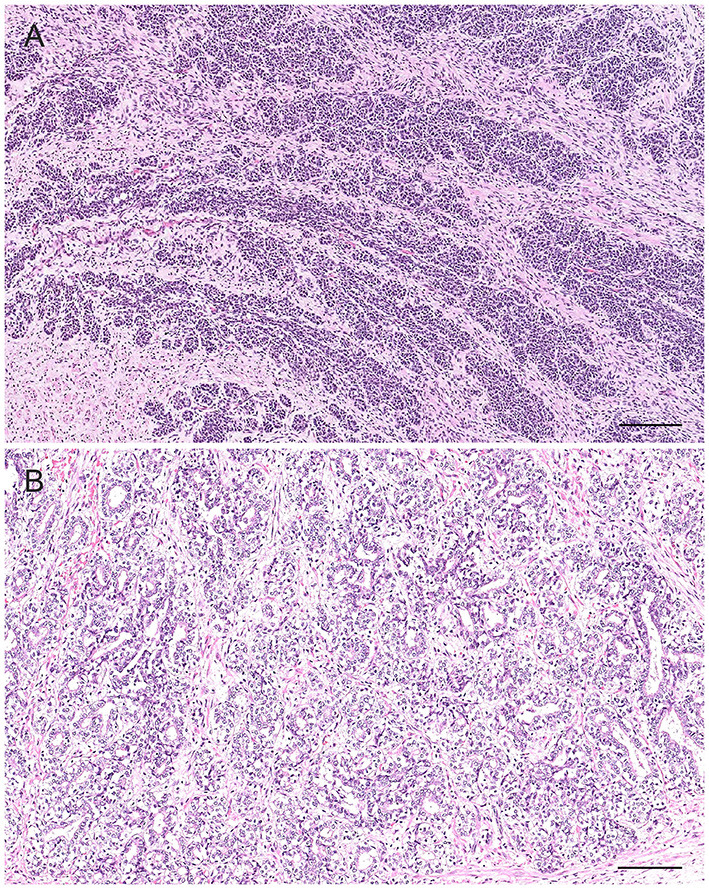
Normal prostate gland from a sexually immature dog. Tissue section. **(A)** Prostate at 12 weeks of age with radiating branching ducts from the prostatic urethra (not visible; on the right-hand side). Hematoxylin-eosin staining. Bar = 400 μm. **(B)** Prostate at 24 weeks of age: irregular glands with a small lumen and moderate amount of stroma. Hematoxylin-eosin staining. Bar = 200 μm.

There is a marked development of the canine prostate after birth between 24 and 32 weeks of age reflecting the increase in number of Leydig cells in the testis after 28 weeks of age and increased plasma testosterone levels (5, 6). The prostate from sexually immature dogs consists of a branching ductular system radiating from the prostatic urethra admixed with not well-developed acini scattered in the external portion of the gland. These small glandular structures lack any lumen until 8 weeks of age. Most of the prostatic tissue is occupied by connective tissue stroma (7). Between 24 and 32 weeks of age, there is a progressive increase in number and size of the glandular alveoli, increased height of the glandular epithelium, formation of small epithelial projections within the lumen and intraluminal accumulation of proteinaceous material that marks the beginning of the secretory function of the prostatic epithelium (7) (Figures 1A,B). Sexual maturity in dogs varies according to the breed and even according to the bodyweight within the same breed (6, 8). Therefore, it is recommended to examine the testes together with the prostate to determine the age of sexual maturity. This may be extremely important for toxicologic studies since immature acini could lead to a misdiagnosis of treatment-related effect of glandular atrophy, while it may just be expression of the stage of sexual maturity.

About 63% of dogs develop progressive enlargement of the prostate with age after puberty (9). On the other side, following castration, the canine prostate undergoes an involution because of androgen deprivation with the serum testosterone concentration reaching very low baseline values already in the 1 week post-castration (10). A 70% reduction of the size of the organ is already obvious 7–14 days after castration and this is likely to occur also in cats (11, 12).

**Figure 2:**
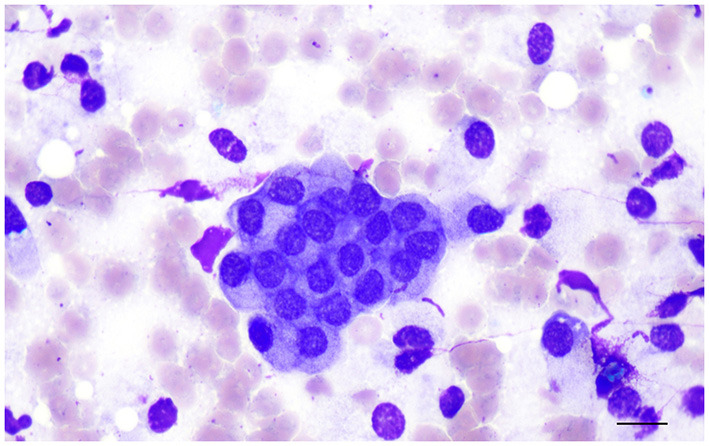
Prostate aspirate showing normal prostatic epithelial cells in small cluster with centrally located nuclei, finely stippled or reticular chromatin pattern and finely granular cytoplasm. Prostate fine needle aspirate (FNA), smear. Wright-Giemsa stain. Bar = 12 μm.

Material from the prostate for cytological evaluation may be obtained through the urethra (prostatic massage), ejaculation or direct fine needle aspirate (FNA) (13, 14) and the collection technique influences the morphology of the epithelial cells and the number of other cell types in the cytological samples. Normal prostatic epithelial cells obtained from aspiration from normal dogs usually occur in small to medium clusters, are uniform in shape and size, cuboidal to lowly columnar with small to moderate amount of occasionally vacuolated basophilic cytoplasm, round to oval central nuclei with a reticular chromatin pattern and small to inconspicuous nucleoli (Figure 2). Individual or small cluster of mostly round epithelial cells with the same nuclear and cytoplasmic features as described above are instead observed in cytological samples collected by prostatic massage. Other cell types that may be present are spermatozoa, squamous epithelial cells and urothelial cells (13, 14). The number of other cell types in cytologic samples depends on the collection technique: spermatozoa are most frequently found in ejaculated material, while squamous cells deriving from the distal urethra or the external genitalia and urothelial cells can be found in samples obtained by both prostatic massage and ejaculation (13, 14).

**Figure 3:**
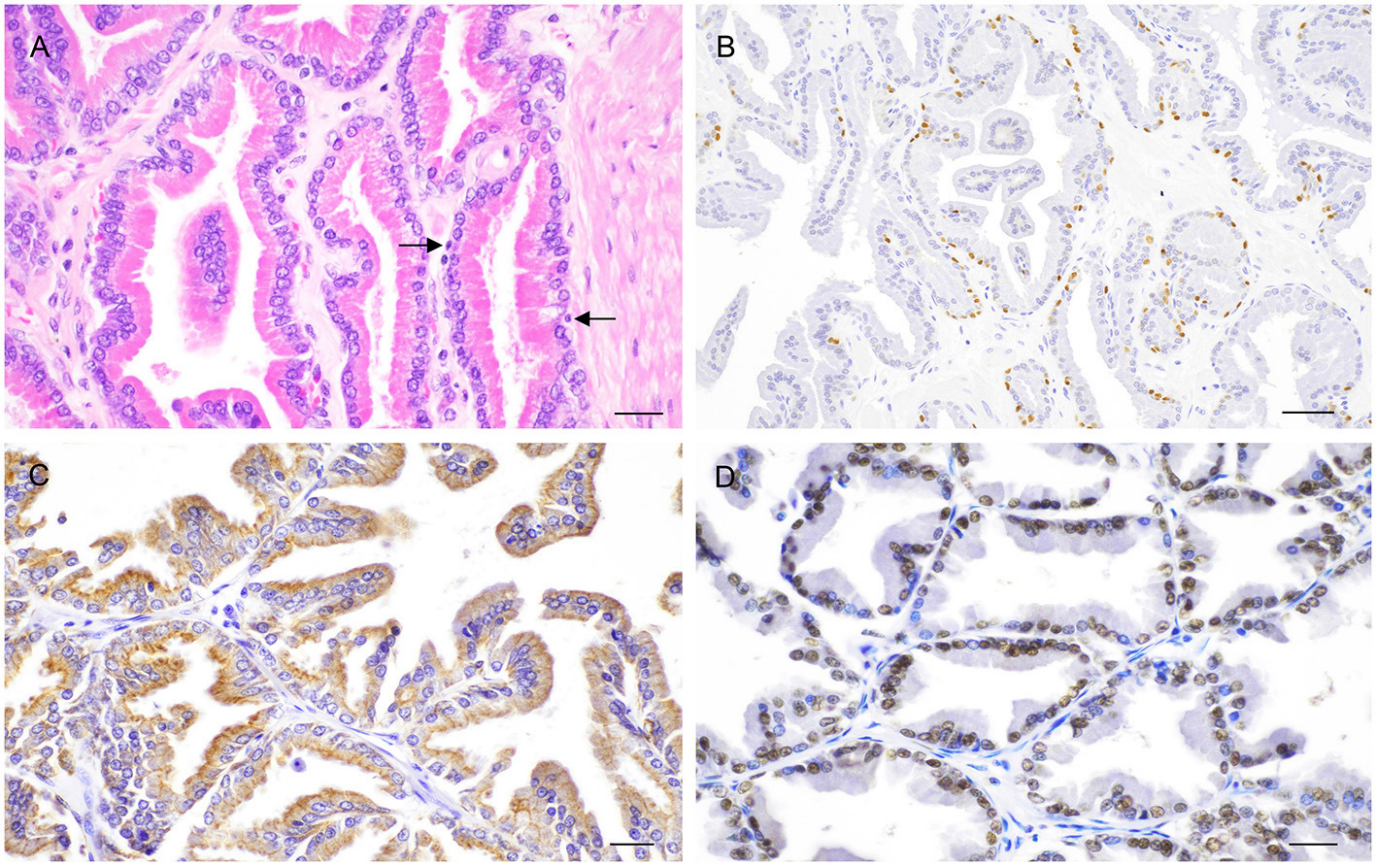
Histological and immunohistochemical features of the normal canine prostate. **(A)** Tubuloalveolar gland lined by secretory columnar cells with low number of basal cells (arrows). Hematoxylin-eosin staining. Bar = 25 μm. **(B)** Nuclear expression of p63 by basal cells. IHC. DAB chromagen. Meyer's hematoxylin counterstain. Bar = 100 μm (mouse monoclonal anti-mouse p63, Dako, 1:150). **(C)** Cytoplasmic expression of CK8/18 by columnar secretory cells. IHC. DAB chromagen. Meyer's hematoxylin counterstain. Bar = 25 μm (mouse monoclonal anti-human Ck8/18, Novocastra, 1:600). **(D)** Nuclear positive staining for androgen receptor (AR) by columnar secretory cells. IHC. DAB chromagen. Meyer's hematoxylin counterstain. Bar = 25 μm (rabbit polyclonal anti-human AR, Santa Cruz Biotech., 1:1,000).

Histologically, the glandular portion of the prostate consists of tubuloalveolar glands producing secretions that are conveyed to the prostatic urethra through small periurethral ducts. Small ducts are also present in the periphery of the prostate, but they cannot be easily discerned without specific staining. The normal epithelium of the prostate consists of two cell layers: a luminal or secretory cell layer and a basal cell layer (Figure 3A). A third cell type in the normal human prostatic epithelium is the neuroendocrine (NE) cell (15), whose existence in cats and dogs is controversial (16, 17). In two of the most recent studies on identification of NE cells in dogs and cats, rare serotonin-positive cells have been described in normal and hyperplastic canine prostates with their number increasing after castration (18) and serotonin—and chromogranin A-positive cells in the prostate of sexually mature healthy cats (19). Basal cells are scarce, round to oblong, but occasionally flattened, with scant amount of dense eosinophilic cytoplasm and a small hyperchromatic nucleus. Small nucleoli may be occasionally seen. The immunophenotype of basal cells is distinctive and can be of diagnostic utility. Antibodies directed against high molecular weight cytokeratin (HMWK), cytokeratin 5 (CK5), and p63 react with basal cells (Figure 3B) while the same cells are mostly PSA (Prostate Specific Antigen)-, AR (Androgen receptor)-, and CK8/18-negative (20–22). Basal epithelial cells form a discontinuous layer in the canine prostate, while the lack of a continuous basal cell layer in humans is a strong indicator of prostatic carcinoma (23). The precise function of normal prostatic basal cells is unclear in dogs, although it is likely that the basal cell population harbors stem cells (24). The secretory or luminal cells make up the bulk of the epithelial volume. They are cuboidal to columnar with nuclei in the basal or midportion of the cell, an hyperosinophilic granular cytoplasm with occasional vacuolations, and small round nuclei with fine evenly dispersed chromatin. Nucleoli are not usually evident or are pinpoint in size. Secretory cells are PSA-, CK8/18-, and AR-positive (20–22) (Figures 3C,D). The columnar cells of the prostatic acini gradually change to a single lining of cuboidal epithelial cells within the ducts.

## Canine Prostate Pathology

Although large-scale epidemiologic studies on the prevalence of prostatic disorders are lacking, prostatic hyperplasia and acute or chronic prostatitis are definitely the most common lesions observed in dogs with an incidence of 46–55.3% and 28–38.5% respectively. These are followed by prostatic cysts (2.6–14%) and prostatic neoplasia (0.2–0.35%) (25–30).

### Prostatic Hyperplasia

Prostatic hyperplasia (PH)—traditionally called benign prostatic hyperplasia (BPH)—is the most common prostatic disorder in intact dogs. Approximately 50% of dogs may have histologic changes of PH by 4–5 years of age and more than 90% by 8 years of age (31). Since the term hyperplasia is already defining a benign process, it is preferable to use PH instead of BPH, as recommended by the canine prostate cancer subgroup of the Oncology Pathology Working Group (OPWG), a joint initiative of the Veterinary Cancer Society and the American College of Veterinary Pathologists (32). The estrogen: testosterone ratio is increased in affected dogs. Estrogens enhance androgen receptors and acts synergistically with an overproduction of dihydrotestosterone (DHT) in potentiating the hyperplastic process (33). This explains why the administration of androgens in combination with estrogens to orchiectomized dogs induces prostatic hyperplasia (34).

**Figure 4:**
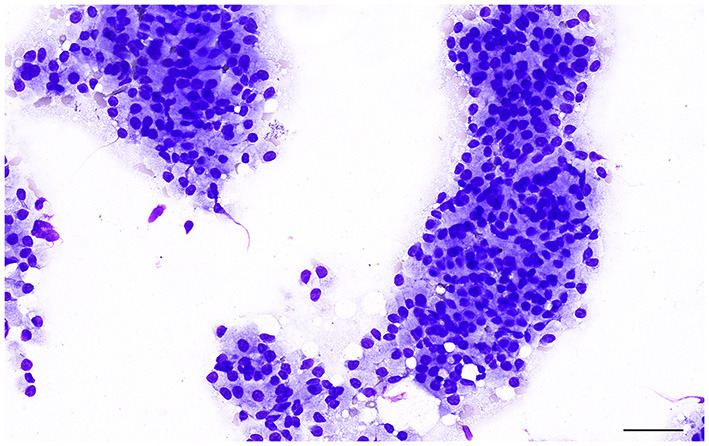
Prostate aspirate showing a large cluster of uniform prostatic epithelial cells from a dog with prostatic hyperplasia. The cells have moderate amount of granular cytoplasm and round, central to eccentric nucleus with finely stippled to reticular chromatin. Note the pink granular material on the background, most likely indicating the secretory proteinaceous activity of the glandular epithelial cells. Prostate FNA, cytospin. Wright-Giemsa stain. Bar = 50 μm.

Clinical signs are present only once the prostate is large enough and affected animals may be presented with urethral discharge, tenesmus, hematuria, fertility abnormalities and occasionally a stilted gait caused by prostatic pain (25, 26, 35). Cytologically, the hyperplastic epithelial cells are very similar to the normal prostatic epithelium, although the cellularity of the sample may be higher (13, 14) (Figure 4). Mild increase in cell size and anisokaryosis may be noted. Cells can exfoliate in large sheets in a honeycomb pattern (13, 14).

**Figure 5:**
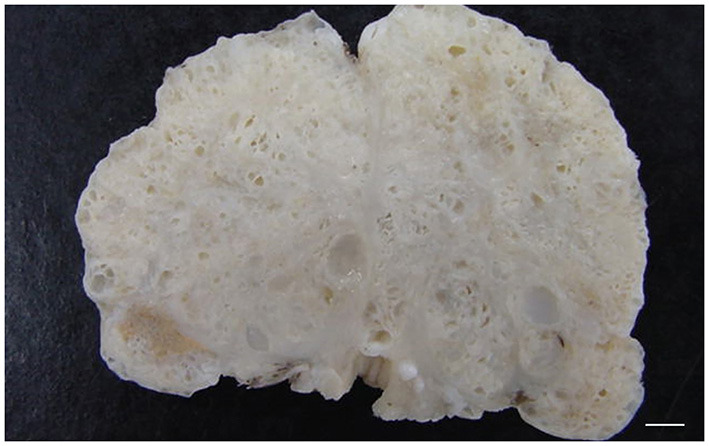
Transverse cross-section of a canine prostate with evidences of prostatic hyperplasia. Enlarged prostate with multifocal small cystic lesions. Bar = 0.35 cm.

**Figure 6:**
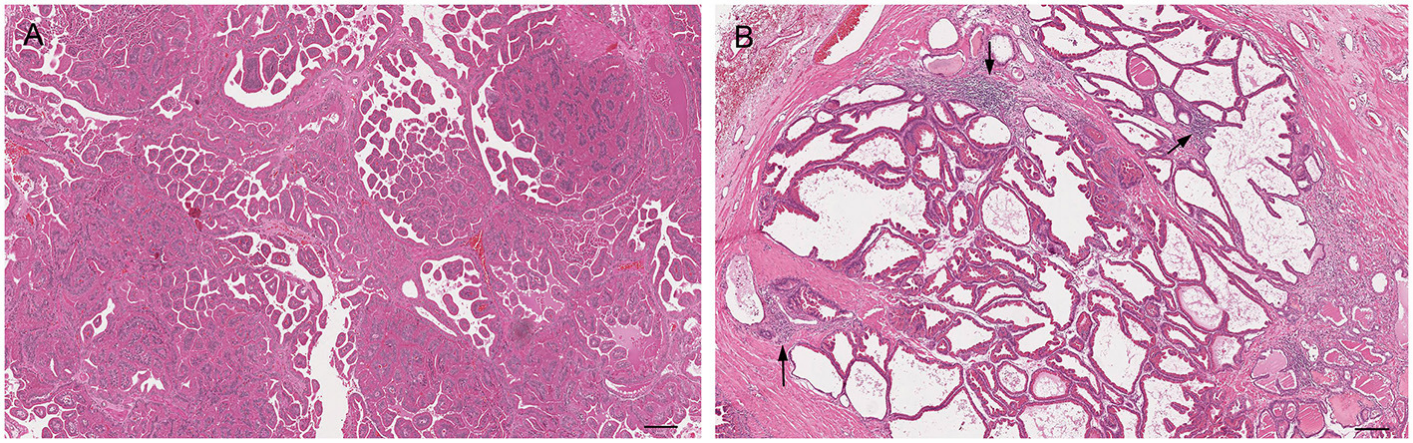
Histological subtypes of prostatic hyperplasia in dogs: **(A)** Glandular hyperplasia: large alveoli with numerous intraluminal papillary projections admixed with a reduced amount of stroma. Hematoxylin-eosin staining. Bar = 100 μm. **(B)** Complex hyperplasia: multifocal lesion with dilated and cystic alveoli. Note the randomly scattered aggregates of inflammatory cells (arrows). Hematoxylin-eosin staining. Bar = 200 μm.

Grossly, PH-affected dogs have a uniformly enlarged prostate and frequently variable-sized cysts (Figure 5). PH is a spontaneous morphologic change associated with aging and sex hormone dysregulation and can be histologically classified into glandular and complex hyperplasia (31, 32, 36). A breed predisposition (Rhodesian ridgeback) has been reported by Beining et al. (37), although this finding should be further investigated and confirmed in a large-scale study. Glandular hyperplasia occurs in dogs as early as 2–3 years of age and consists of a uniform glandular enlargement of the prostatic alveoli, increased papillary infoldings and increased number of columnar secretory cells (7, 38) (Figure 6A). The hyperplastic epithelial cells have basal nuclei and prominent granules in the apical cytoplasm. It is important to emphasize that early hyperplastic changes are histologically indistinguishable from a normal prostate and the weight of the gland relative to the age, bodyweight and breed may help in most but not all cases. Complex hyperplasia occurs in dogs older than 6 years of age and is characterized by the presence of cystic lesions admixed with areas of glandular hyperplasia and atrophic glands, separated by an increased amount of stroma (collagen, smooth muscle) (36) (Figure 6B). Interestingly, DeKlerk et al. (38) induced canine PH in Beagle dogs by administering both 17β-estradiol and either 5α-androstane-3α, 17β-diol or dihydrotestosterone. Young dogs developed glandular hyperplasia, while cystic/complex hyperplasia was never observed in this age group (38). Therefore, aging represents an important contributing factor for the development of cystic hyperplasia. In some cases of PH, chronic inflammation with infiltration of mononuclear cells may be present (5, 38) (Figure 6B).

### Prostatitis

**Figure 7:**
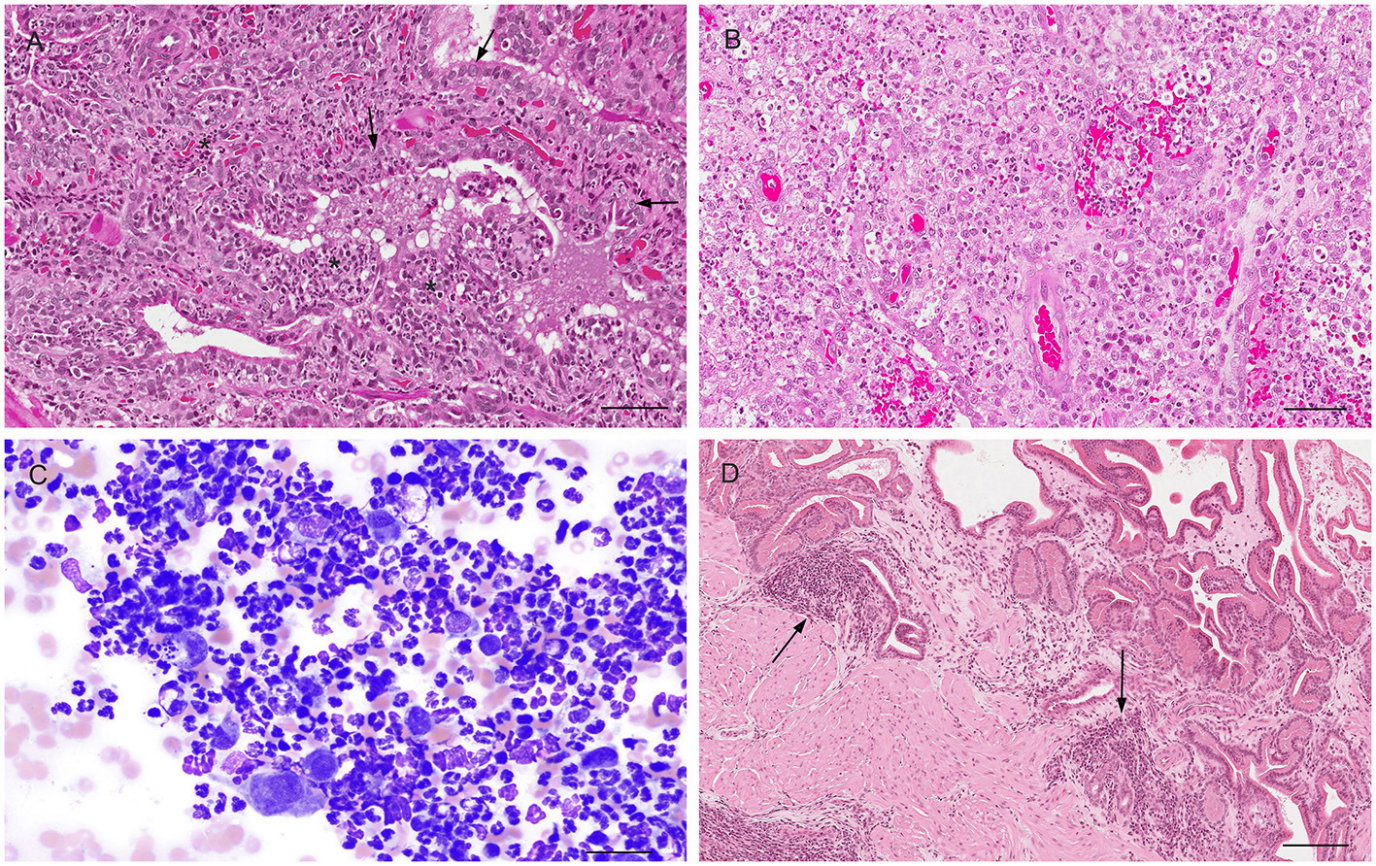
Histological **(A,B,D)** and cytological **(C)** features of three different types of prostatitis in dogs **(A)**. Acute prostatitis with infiltration of high number of neutrophils (asterisks) and occasional reactive changes in the glandular epithelial cells (large nuclei, prominent nucleoli) (arrows). Hematoxylin-eosin staining. Bar = 60 μm. **(B)** Infiltration of high number of neutrophils and macrophages with severe destruction of the gland (pyogranulomatous prostatitis). Hematoxylin-eosin staining. Bar = 60 μm. **(C)** Prostate aspirate showing cytological features of acute prostatitis with high number of neutrophils with minimal nuclear degeneration and rare prostatic epithelial cells. Prostate FNA, cytospin. Wright-Giemsa stain. Bar = 30 μm. **(D)** Mild multifocal chronic lymphoplasmacytic prostatitis (arrows). Hematoxylin-eosin staining. Bar = 120 μm.

Prostatitis is a common histologic finding in prostates of intact dogs. Reports in castrated dogs are exceptionally rare and usually associated with a history of recent castration before presentation. This inflammatory process may be subclinical or clinically relevant, especially in the acute stage with the infiltration of large number of neutrophils within the interstitium and in the lumen of the glandular acini (32, 39) (Figure 7A). These acute inflammatory forms are usually caused by *Escherichia coli* or *Proteus vulgaris* ascending from the urethra. Other common isolates are *Staphylococcus spp., Streptococcus spp., Pseudomonas spp., Klebsiella spp., Enterobacter spp., Pasteurella, Haemophilus* (11, 33, 40). Other inflammatory cells can also be identified in histological sections such as macrophages, lymphocytes, and plasma cells either in the prostatic acini or stroma (32, 39) (Figure 7B). According to the severity of the infection, additional histologic findings may be interstitial edema, hemorrhage and/or necrosis. Cytological samples from cases of bacterial prostatitis contains high number of neutrophils (Figure 7C), mostly with degenerative changes, exfoliated hyperplastic prostatic epithelial cells, intracellular and extracellular bacteria (in the absence of previous antibiotic therapy) and occasional macrophages and lymphocytes, especially if the infection is becoming chronic (13, 14). Acute prostatitis is usually associated with fever, anorexia, depression, straining to urinate or defecate, caudal abdominal pain, hematuria, pain on rectal palpation and sporadically edema of the scrotum, prepuce and hindlimb (41).

Prostatitis may be also observed with *Brucella canis* infection (chronic interstitial prostatitis) (42), dissemination of fungal organisms through a systemic infection (*Blastomyces dermatitis, Cryptococcus neoformans, Coccidioides immitis*) causing a granulomatous prostatitis (40, 43, 44) and rarely *Mycoplasma canis* and *Leishmania* spp. (45, 46). On the other hand, chronic prostatitis might occur in dogs without any clinical signs. When present, clinical signs of chronic prostatitis are non-specific, with affected dogs experiencing recurrent urinary tract infections, poor semen quality, infertility, decreased libido, intermittent urethral discharge (41). Histopathology reveals focal or multifocal infiltration of mononuclear cells in the prostatic stroma and variable degree of atrophy of the prostatic secretory cells and fibrosis (32) (Figure 7D).

### Prostatic Abscess

Prostatic abscess is usually considered a sequela of chronic prostatitis (26) or can occur in association with acute bacterial prostatitis and cystic hyperplasia (40). Rarely, prostatic abscess may be secondary to bacteremia (26, 28). The diagnosis is usually made by history, clinical examination, ultrasonography of the prostate, laboratory findings and bacterial culture of the urine and/or prostatic fluid (40). Clinical signs may be variable depending on the size of the lesion and whether the infection becomes systemic. They include tenesmus and dysuria caused by the progressive enlargement of the prostate or urethral discharge or systemic symptoms caused by endotoxemia (33). Histologically, prostatic abscesses are characterized by a severe neutrophil infiltration, with gland destruction and necrosis.

### Prostatic Cystic Conditions

Prostatic and paraprostatic cysts are grouped under the same paragraph as conditions characterized by the presence of cystic lesions, although they have different pathogenesis and distribution. Clinical signs are uncommon, unless the size of the cyst becomes large enough to cause dysuria, tenesmus and hematuria (33). Cytologically, they may be acellular or containing few normal epithelial cells, rare non-degenerative neutrophils, macrophages, small lymphocytes and/or erythrocytes and cellular debris (13, 14). Prostatic cysts are traditionally considered a further evolution of a cystic PH with grossly detectable cysts within the prostatic parenchyma and a classical “honeycomb” appearance (26) (Figure 5). Histologically, prostatic cysts are large acini with or without intraluminal proteinaceous material lined by atrophic glandular cells. Intraprostatic cysts may also occur in association to squamous metaplasia and prostatitis or when ducts are obstructed leading to accumulation of prostatic fluid, hence the definition of prostatic retention cysts. Some paraprostatic cysts arise from a cystic uterus masculinus, a remnant of the paramesonephric duct, and are commonly located in the craniolateral or dorsal aspect of the prostate (47). Grossly, they are large nodular structures, usually palpable through the pelvic cavity (they can reach up to 30 cm in diameter), enclosed within a fibrocollagenous capsule that might undergo ossification and mineralization (26, 48). Mineralization of paraprostatic cyst is reported to be uncommon (49–51), although Renfrew et al. (52) have observed mineralized cysts in 3 out of 6 affected dogs, concluding that this process is actually more common than implied in the literature.

### Prostatic Atrophy

**Figure 8:**
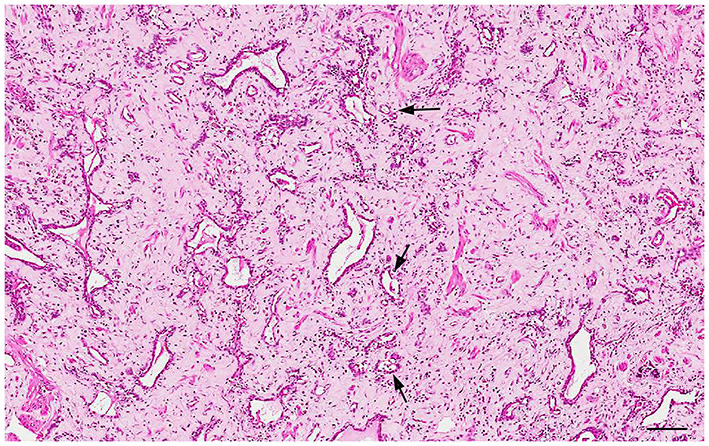
Histology of the prostate from a castrated dog: hormonal atrophy with relative increase of the interstitial stroma and tubules lined by a single epithelial layer. Hematoxylin-eosin staining. Bar = 120 μm.

**Figure 9:**
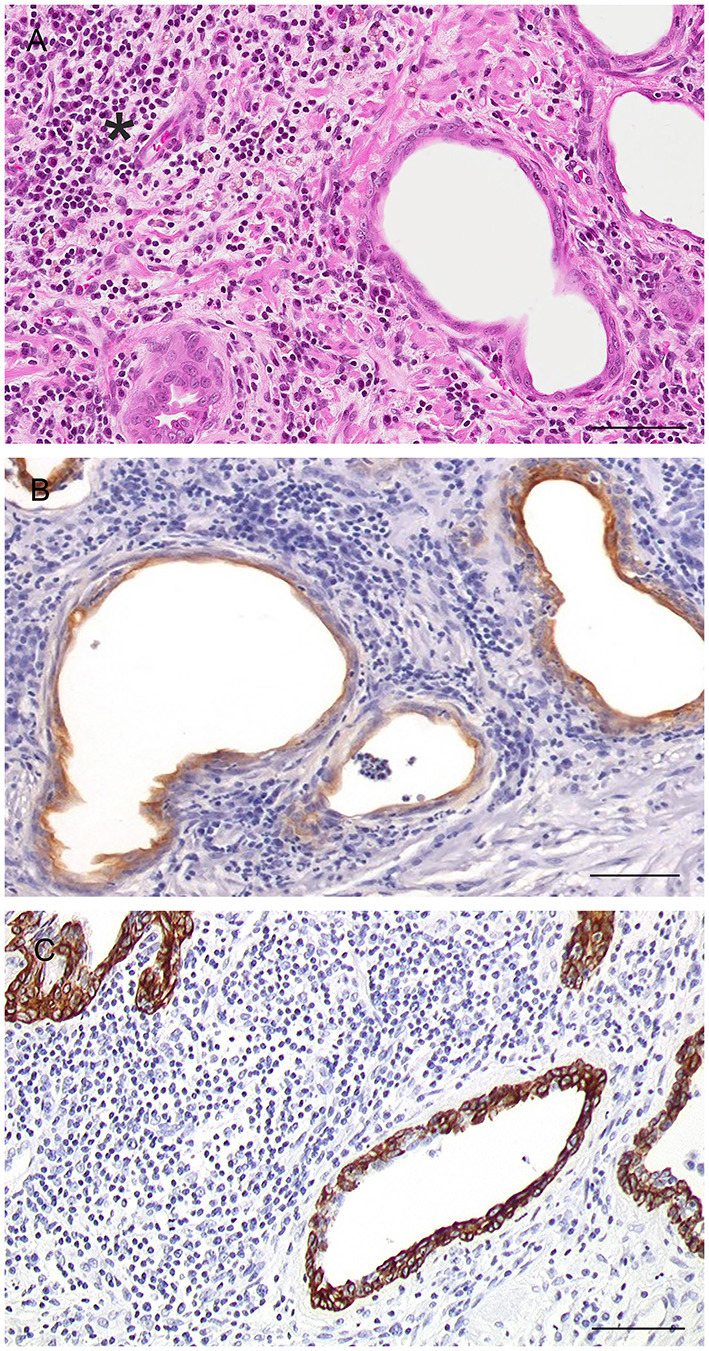
Histological and immunohistochemical features of glandular atrophy associated with chronic inflammation (PIA-like lesions). **(A)** Gland with attenuated epithelium and surrounding chronic inflammatory infiltrate (asterisk). Hematoxylin-eosin staining. Bar = 120 μm. **(B)** Cytoplasmic expression of CK8/18 by epithelial cells of the PIA-like lesions. IHC. DAB chromagen. Meyer's hematoxylin counterstain. Bar = 120 μm (mouse monoclonal anti-human Ck8/18, Novocastra, 1:600). **(C)** Cytoplasmic expression of CK5 by epithelial cells of the PIA-like lesions. IHC. DA chromagen. Meyer's hematoxylin counterstain. Bar = 120 μm (mouse monoclonal anti-human CK5, Novocastra, 1:300).

Prostatic atrophy in dogs can be classified into atrophy secondary to neutering (hormonal atrophy) and atrophy associated with chronic inflammation (32). The hormonal atrophy has no clinical significance and may also be secondary to the administration of antiandrogenic drugs (32) and GnRH agonists and antagonists (53). Castration induces a progressive shrinkage of the prostatic acini with relative increase of the fibromuscular tissue until only low number of tubules with a single epithelial lining remains in the advanced stage (54) (Figure 8). On the other hand, prostatic atrophy can occur in intact or castrated dogs in association with a focal, multifocal or diffuse lymphoplasmacytic inflammatory infiltrate. At low magnification, these atrophic glands show a hyperchromatic appearance, surrounded by the inflammatory infiltrate. At higher magnifications, the atrophic acini containing at least two layers: (1) basal cell layer and (2) secretory atrophic layer (Figure 9A). Both cell populations have reduced cytoplasm and hyperchromatic nuclei. Although these cells are morphologically atrophic, they are also proliferative (“paradox” atrophy) and this specific lesion is defined as Proliferative Inflammatory Atrophy (PIA) in humans. PIA is considered a precursor of Prostatic Intraepithelial Neoplasia (PIN) and prostate cancer (55). Although there are still no studies demonstrating a strict correlation between PIA and progression to cancer in dogs, PIA-like lesions have been described in dogs, both in normal and neoplastic prostates (29, 56–58). The cell population of these lesions in dogs show an intermediate phenotype, expressing both luminal and basal cell markers (59) (Figures 9B,C).

### Squamous Metaplasia

**Figure 10:**
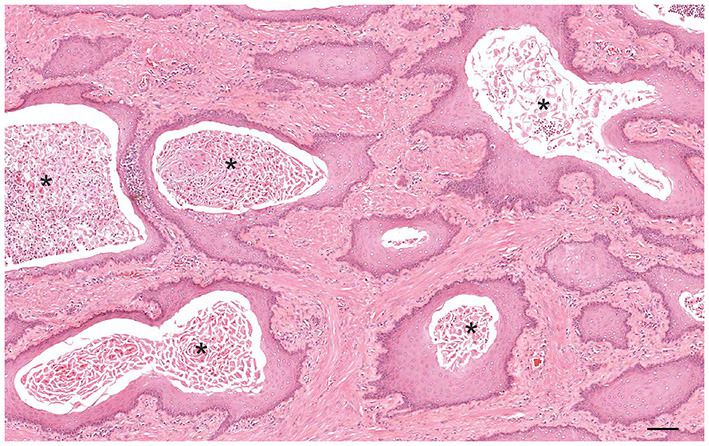
Histology of squamous metaplasia in a dog with Sertoli cell tumor. The glandular alveoli are lined by a pluristratified squamous epithelium with intraluminal accumulation of keratin squames and sloughed epithelial cells (asterisks). Hematoxylin-eosin staining. Bar = 200 μm.

When the prostate undergoes squamous metaplasia, the columnar glandular epithelium becomes stratified squamous, with keratin squames shed into the lumen together with sloughed epithelial cells (Figure 10). The metaplastic epithelium may be observed in the glandular acini only or even in the ducts. Inflammatory cells (degenerated neutrophils and/or macrophages) may be also present in the lumen, especially if the metaplastic lesion is secondary to chronic prostatitis. In the latter case, squamous metaplasia is a very subtle change and usually a focal finding.

Cytological samples contain aggregates of large, angular and flattened, well-differentiated squamous epithelial cells with abundant pale to light blue cytoplasm and a central, pyknotic or karyorrhectic nucleus. Squamous cells may be admixed with inflammatory cells, bacteria or hyperplastic prostatic cells according to the underlying disease process (13, 14).

This lesion may occur spontaneously in association with the estrogen-producing Sertoli cell tumor (60) or following the administration of estrogens (61). Clinical signs usually related to hyperestrogenism. Short-term exposure to estrogens causes metaplastic changes in the periurethral duct tissue, while changes in the entire gland are secondary to long-term administration of estrogens (62). Interestingly, metaplastic prostates return to the normal morphology once the estrogen stimulation is removed (63, 64). It is likely that estrogens induce proliferation of the basal cells with squamous differentiation rather than androgen-driven differentiation into secretory cells (54). Squamous metaplasia of the canine prostate is not considered a pre-neoplastic change, but it can lead to the formation of cysts and/or abscesses.

### Prostatic Tumors

Prostatic tumors are rare in dogs, with variable incidence according to the studies. The lack of specific markers for prostatic cancer in dogs as well as effective diagnostic screening tests makes early diagnosis difficult and therefore the issue of underestimation of cases in the canine population is real.

Prostatic carcinoma is considered androgen-independent and metastasize rapidly in ~70–80% of cases. Reported sites of metastases are regional lymph nodes, pelvic musculature, vertebral bodies, lung, liver, urethra and urinary bladder, colon and rectum, spleen, heart, kidney, distant lymph nodes and adrenal glands (36). Although there is a common belief that there is an increased risk of carcinoma in castrated dogs (65, 66), carcinoma of the prostate occurs with the same prevalence in sexually intact and neutered dogs (67–71).

Many dogs do not show any clinical signs until late in the course of the disease. When present, the most common clinical signs—excluding those related to potential metastases—are prostatomegaly, painful abdominal palpation, stranguria, dysuria, hematuria, constipation, tenesmus, anorexia, weight loss, pain and paresis of hind limb (26, 28).

**Figure 11:**
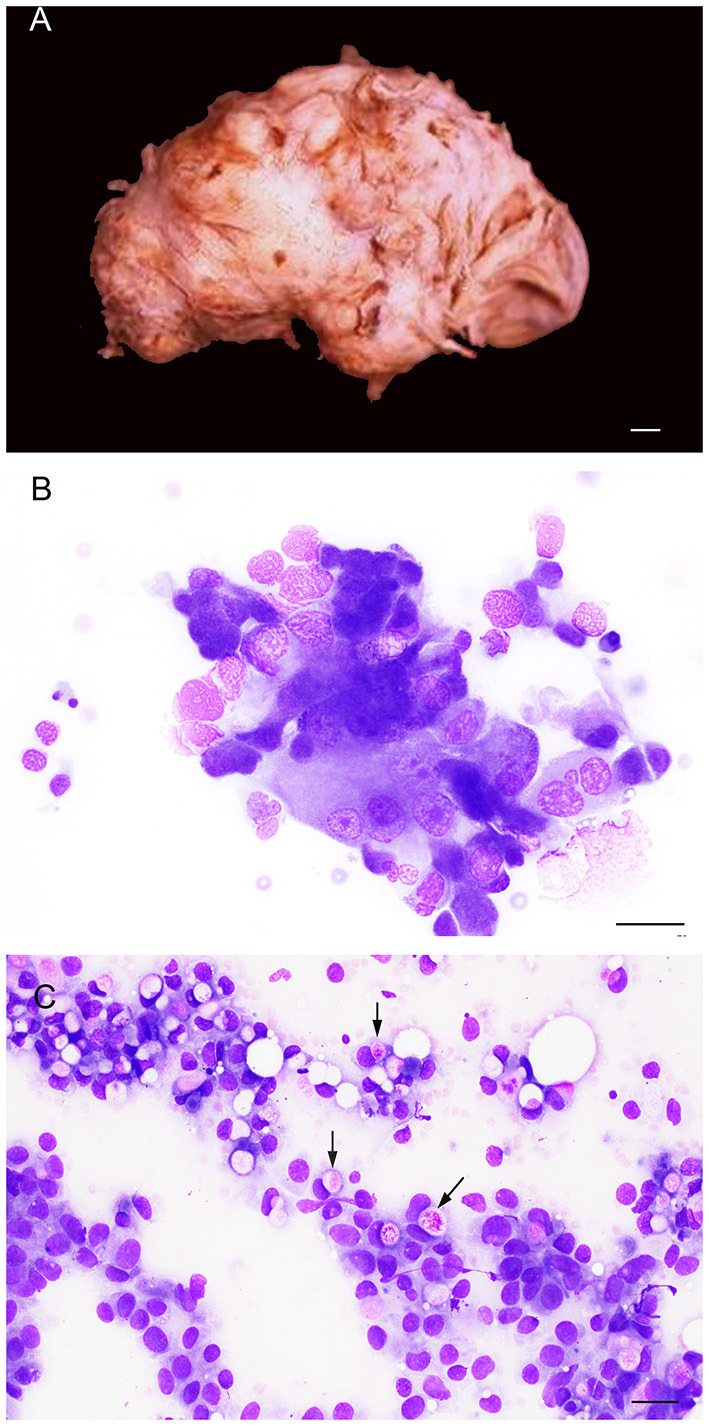
Gross **(A)** and cytological **(B,C)** features of prostatic carcinoma in dogs. **(A)** The prostate is irregularly enlarged, showing a multinodular gross aspect (entire prostate removed from the connections with the urinary bladder and penile urethra). Bar = 1 cm. **(B)** Prostate aspirate showing a small group of neoplastic cells with cytological features of malignancy (pleomorphism, large nuclei, multiple prominent nucleoli). Prostate FNA, smear. Wright-Giemsa stain. Bar = 25 μm. **(C)** Prostate aspirate showing neoplastic epithelial cells from a urothelial carcinoma of the prostate with intracytoplasmic vacuoles containing bright pink material (arrows). Prostate FNA, smear. Wright-Giemsa stain. Bar = 20 μm.

Grossly, the prostate may be asymmetrically and irregularly enlarged (Figure 11A), with or without invasion of the surrounding organs. In other cases, the prostate present little changes and only a slight enlargement may be detected.

Canine prostatic carcinoma is classified into prostatic adenocarcinoma (AC) and prostatic urothelial carcinoma (UC), although a mixed phenotype can also occur (32). In classical cases, cytological samples consist of variably sized clusters, sheets or scattered single cells with marked criteria of malignancy, including single to multiple nuclei, coarse to clumped chromatin, multiple and prominent nucleoli, severe anisocytosis and anisokaryosis, nuclear molding (Figure 11B). Mitoses are common and may be bizarre (13, 14). Prostatic adenocarcinoma and prostatic urothelial carcinoma may be difficult to distinguish cytologically. Some acinar structures may be observed in AC, while urothelial cells show tailed shapes and sporadic cytoplasmic vacuoles with bright-pink material (Figure 11C), although these features may not be consistently present in all cases and are not pathognomonic for UC (13, 14).

**Figure 12:**
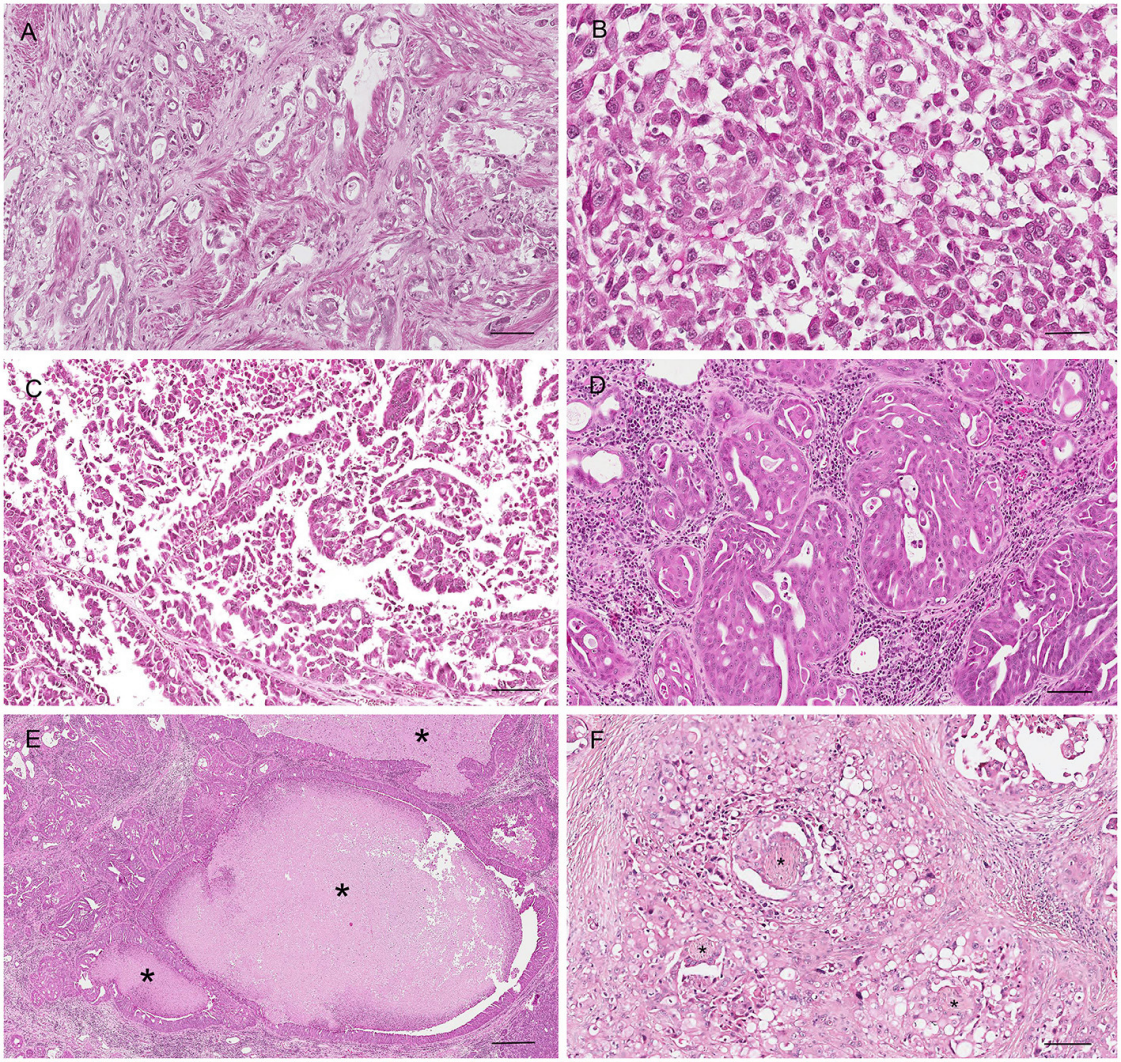
Histological subtypes of canine prostatic carcinoma. **(A)** Tubular carcinoma: small tubules lined by neoplastic epithelial cells. Hematoxylin-eosin staining. Bar = 120 μm. **(B)** Solid carcinoma: pleomorphic neoplastic cells arranged in sold nests without a glandular structure. Hematoxylin-eosin staining. Bar = 40 μm. **(C)** Papillary carcinoma: neoplastic cells forming papillary projection within the lumen. Hematoxylin-eosin staining. Bar = 120 μm. **(D)** Cribriform carcinoma: neoplastic cells filling the lumen with the formation of regular fenestrae. Bar = 120 μm. **(E)** Cribriform carcinoma with central area of necrosis (comedonecrosis) (asterisks). Hematoxylin-eosin staining. Bar = 500 μm. **(F)** Perineurial invasion: neoplastic cells encircle and proliferate around nerves (asterisk). Hematoxylin-eosin staining. Bar = 120 μm.

Prostatic adenocarcinoma (AC) originates from the glandular epithelium and displays a high degree of morphological heterogeneity in terms of growth patterns and histological subtypes, occurring individually or in combination. The simple tubular pattern is characterized by the formation of small acini and tubules (Figure 12A). The solid pattern is composed of solid sheets, cords or isolate pleomorphic epithelial cells, that can occasionally be spindle shaped (Figure 12B). In the papillary subtypes, cuboidal to columnar cells neoplastic cells form papillary projections with a delicate fibrovascular core within an extended duct (Figure 12C). Cribriform prostatic adenocarcinoma shows irregular fenestrated proliferation of the tumor cells filling the lumen of the gland (Figure 12D), usually with central necrotic debris (comedonecrosis) (Figure 12E) (29, 32). Other less common patterns are micropapillary and sarcomatoid (29, 72).

In all the subtypes, the mitotic index is moderate to high, except for the tubular pattern that usually exhibits a low mitotic rate. It is not unusual to find additional histologic features, such as lymphatic invasion, squamous differentiation, perineurial invasion (Figure 12F) (29, 32).

Prostatic urothelial carcinoma (UC) arises from the urothelial cells of the prostatic urethra or the periurethral ducts. Neoplastic cells are extremely heterogeneous, ranging from small polyhedral to large cells, with low to abundant eosinophilic cytoplasm, hyperchromatic or large vesicular nucleus and high mitotic index. Melamed-Wolinska bodies—intracytoplasmic eosinophilic bodies—can be an important morphologic feature that helps in the diagnosis of urothelial cell tumors (32).

Distinction between urothelial and prostatic origin can be difficult in most cases, except with the tubular histotype that is more aligned with a diagnosis of prostatic adenocarcinoma. The location of the tumor, close to the prostatic urethra, favors a urothelial origin. Neoplastic cells with cytoplasmic vacuoles (signet cells) and absence of tubules and acini would be more supportive of a UC, although signet cells can also be present in areas of urothelial metaplasia within a prostatic adenocarcinoma. Immunohistochemistry (IHC) can be performed if needed (uroplakin III, CK7), although a definitive marker to differentiate urothelial from glandular origin of the tumor is still lacking. It is common practice to rule in a urothelial carcinoma when IHC reveals immunoreactivity of the neoplastic cells to UPIII and CK7 (73). However, cytokeratin 7, previously thought to be expressed by urothelial cells of the prostatic urethra and periurethral prostatic ducts only (74), is actually found even in the glandular epithelium, so that positive staining can be similarly observed in AC and UC (75). Uroplakin III is more sensitive and specific for urothelial tumors in dogs, although it may be negative in 10% of cases, the staining is not uniform within the same tumor and positive regions may be missed and it is consistently negative in undifferentiated tumors (73). As an additional complicating factor, some tumors show a combination of prostatic adenocarcinoma and urothelial carcinoma (73, 75).

Prostatic squamous cell carcinoma shows closely packed non-keratinizing epithelial cells with moderate to large eosinophilic cytoplasm and occasional prominent intracellular bridges and keratin pearls. Primary prostatic squamous cell carcinoma should be differentiated from urinary bladder squamous carcinoma invading the prostate or prostatic urothelial carcinoma with squamous differentiation.

Mesenchymal prostatic tumors, such as fibrosarcoma, leiomyosarcoma, hemangiosarcoma can occur, with features similar to other organs (76–78). Lymphoma has also been described in the canine prostatic gland, mainly in the disseminated disease (79, 80).

In humans, prostate carcinoma may evolve from a well-defined and characterized lesion, called prostatic intraepithelial neoplasia (PIN), that defines a proliferation of atypical epithelial cells within preexisting ducts and acini (15). High-grade PIN (HGPIN) lesions have been described in the prostate of dogs with and without prostate cancer (80–83). Typical HGPIN consists of glands with nuclear crowding and stratification, luminal cells with enlarged nuclei, large and prominent nucleoli and size and shape variation (84). A critical review of the literature describing these lesions in the canine prostate (32) have evidenced the possibility that these lesions may be or (a) expression of a retrograde invasion of the normal glands by adjacent neoplastic glands; or (b) reactive lesions secondary to an inflammatory reaction in the surrounding stroma or (c) atypical/dysplastic changes. Therefore, a certain degree of caution should be applied when reporting these lesions in a diagnostic setting.

### Prostatic Calculi, Corpora Amylacea and Polyglucosan Bodies

In the human prostate, prostatic calculi are usually associated with inflammation, prostatic hyperplasia or prostatic cancer (15), while this correlation has not been demonstrated in dogs yet. Prostatic calculi can be infrequently seen in the canine prostate as an incidental finding (85, 86). They can originate from the bladder through the prostatic urethra and then the prostatic duct system (exogenous calculi) or form within the prostate itself, usually within a cyst (endogenous calculi). Exogenous calculi contain constituents of the urine, while endogenous calculi can be formed by the precipitation of element present in the prostatic secretions (87).

Corpora amylacea are eosinophilic inspissated secretions that usually assume characteristic concentrical lamellations (15). They are extremely common in the normal human prostate gland, while they are rarely seen in the dog prostate. They can calcify and contributes to the formation of prostatic endogenous calculi. PAS-positive polyglucosan bodies have been described in the smooth muscle cells of the canine prostatic stroma by Kamiya et al. (88), most commonly in aged animals.

## Feline Prostate Pathology

### Prostatic Tumors

Prostatic disorders in cats are exceedingly rare, and, despite the lack of studies on the prevalence of prostatic lesions in cats, prostatic carcinoma is the most commonly reported lesion in case descriptions available in the literature (89–95). Affected cats are 6–11 years old and are usually presented with hematuria, dysuria, stranguria, acute urinary obstruction, urinary incontinence, occasionally constipation, inappetence, weight loss and lethargy. Prostatic carcinoma has been described both in intact (91, 94, 95) and neutered (89–93, 96) animals without a specific breed predisposition (domestic shorthair, mixed breed, domestic longhair, Siberian) (97). Definitive risk factors have not been identified due to the sparse number of reports and lack of epidemiologic data. Prostatic carcinoma in cats has not been classified in any histologic subtypes, although most cases are characterized by high degree of cellular pleomorphism, occasional scirrhous reaction, acinar or solid or tubular growth pattern, high mitotic rate and lymphovascular invasion. One case of sarcomatoid carcinoma with neoplastic epithelial cells arranged in acini and tubules surrounded by haphazardly arranged pleomorphic spindle cells has been described by Zambelli et al. (93).

### Prostatitis

Bacterial prostatitis is also less common in cats than in dogs, presumably due to the uncommon occurrence of other predisposing factors, such as primary prostatic hyperplasia and squamous metaplasia. In addition, most of all the cases of bacterial prostatitis begin secondary to the invasion of pathogens in the urinary tract (98), but bacterial urinary tract infections occur much less frequently in cats than in dogs, with only 1–2% of cats affected during their lifetime (99). Prostatitis in cats may be acute or chronic (100, 101), with predominant neutrophilic or pyogranulomatous inflammation. Occasional abscesses may be observed at gross examination in animals with fever, anorexia, and constipation (102). The most common isolate in cats is *Escherichia coli* (100, 102).

### Squamous Metaplasia

Squamous metaplasia of the prostate with secondary prostatitis has been described in only one cat with interstitial cell neoplasm in a retained testis (103). The histologic features of this lesion are similar to those observed in dogs with prostatic glandular acini lined by multiple layers of squamous epithelial cells.

### Paraprostatic Cyst

Only a single case of paraprostatic cyst has been reported in a 3-year-old neutered male domestic, short-haired cat with a history of pollakiuria. The cyst was lined with transitional epithelium multifocally continuous with the epithelium of the prostatic ducts (104).

## Conclusions

This overview covers all the different disorders affecting the canine and feline prostate providing comprehensive clinical and pathological aspects that may inform pathologists and practitioners in their diagnostic investigation and examination of clinical cases, recognizing the difficulties in making a final diagnosis due to non-specific clinical signs and overlap of different diseases. On the other side, as highlighted by the amount of information included in this review, prostatic diseases are not as infrequent as believed and comparative studies may actually reveal new pathological lesions or pathogenesis that will add another level of complexity to these disorders in domestic animals.

## Author Contributions

CP, CF-A, and RL-A conceived and designed this review, performed literature review and data collection, wrote the manuscript, and reviewed the drafts. CP supervised the project. All authors have read and approved the final submitted manuscript.

## Conflict of Interest

The authors declare that the research was conducted in the absence of any commercial or financial relationships that could be construed as a potential conflict of interest.

## Publisher's Note

All claims expressed in this article are solely those of the authors and do not necessarily represent those of their affiliated organizations, or those of the publisher, the editors and the reviewers. Any product that may be evaluated in this article, or claim that may be made by its manufacturer, is not guaranteed or endorsed by the publisher.
