# Supplementary material for: A Review on Canine and Feline Prostate Pathology
Source: Front Vet Sci. 2022 May 26;9:881232. doi: 10.3389/fvets.2022.881232 (PMC9201985; doi:10.3389/fvets.2022.881232)
Supplement: Supplementary file 1 [file Table_1.DOCX]

**Supplementary material. Details of protocols used for the immunohistochemical characterization of prostatic lesions**

| **Antibody** | **Manufacturer** | **Dilution** | **Detection system** | **Antigen unmasking system** |
| --- | --- | --- | --- | --- |
| Rabbit polyclonal anti-human AR | Santa Cruz Biotechnology | 1:1000 | Avidin-biotin-peroxidase (Vectastain Standard Elite, Vector Labs) | HIAR (microwave, sodium citrate buffer pH 6.0) |
| mouse monoclonal anti-human CK8/18 | Novocastra | 1:600 | Avidin-biotin-peroxidase (Vectastain Standard Elite, Vector Labs) | Proteinase K |
| mouse monoclonal anti-human CK5 | Novocastra | 1:300 | Avidin-biotin-peroxidase (Vectastain Standard Elite, Vector Labs) | HIAR (microwave, sodium citrate buffer pH 6.0) |
| Mouse monoclonal anti-mouse p63 | Dako Cytomation | 1:150 | Peroxidase-conjugated polymer system (Envision, Dako) | HIAR (Pressure cooker, sodium citrate buffer pH 6.0) |

Legend: AR = androgen receptor; HIAR = heat-induced antigen retrieval
